# Conspiracy endorsement and its associations with personality functioning, anxiety, loneliness, and sociodemographic characteristics during the COVID-19 pandemic in a representative sample of the German population

**DOI:** 10.1371/journal.pone.0263301

**Published:** 2022-01-28

**Authors:** Nora Hettich, Manfred E. Beutel, Mareike Ernst, Clara Schliessler, Hanna Kampling, Johannes Kruse, Elmar Braehler

**Affiliations:** 1 Department for Psychosomatic Medicine and Psychotherapy, University Medical Center of the Johannes Gutenberg University Mainz, Mainz, Germany; 2 Else-Frenkel-Brunswik-Institute for Democracy Research, University of Leipzig, Leipzig, Germany; 3 Department for Psychosomatic Medicine and Psychotherapy, Medical Center of the Justus-Liebig University Giessen, Giessen, Germany; 4 Department for Psychosomatic Medicine and Psychotherapy, Medical Center of the Philipps University Marburg, Marburg, Germany; Medical University of Vienna, AUSTRIA

## Abstract

**Background:**

In the context of the COVID-19 pandemic, many individuals have been found to endorse conspiracy beliefs. Socio-demographic variables, personality functioning, anxiety, and loneliness could be risk factors for this endorsement.

**Methods:**

In a representative sample of the German population (*N* = 2,503) measures of conspiracy mentality, conspiracy-related beliefs toward COVID-19, personality functioning (OPD-SQS), anxiety (HADS), and loneliness (UCLA) were assessed. Pearson product-moment correlations and multiple linear regression analyses were conducted.

**Results:**

Conspiracy mentality and conspiracy-related beliefs toward COVID-19 were strongly correlated. Regression analyses found younger age, male gender, lower education, and lower income to be associated with conspiracy mentality. The subscales *relationship model* and *self-perception* of the OPD-SQS were positively related to conspiracy mentality whereas *interpersonal contact* was negatively associated. Higher levels of anxiety were statistically predictive for conspiracy mentality.

**Conclusion:**

Our findings indicate a contribution of personality functioning to the understanding of conspiracy mentality and thus to the advancement of interventions during the pandemic.

## Introduction

In the context of the COVID-19 pandemic conspiracy beliefs have gained increasing (media) attention. As the pandemic confronts societies with an uncontrollable and unprecedented public health challenge and has entailed controversial political choices limiting personal freedoms such as mobility and assembly to contain the pandemic, it might contribute to the endorsement of conspiracies. In general, strong endorsement of conspiracy beliefs is defined as conspiracy mentality which describes a fundamental propensity to assume small, secret but ill-intending and powerful groups of an international conspiratorial network to be responsible for social and political phenomena [1]. This belief offers a pattern of explanation that can be applied to phenomena creating uncertainty or fear [2]. Therefore, conspiracy mentality fulfills psychological needs like providing an illusionary sense of control, reduction of complexity, belongingness to a group endorsing the same beliefs, and narcissistic gratification [3]. Those mechanisms might promote endorsement of conspiracy beliefs during the current pandemic. First results indicated that up to 50% of participants from Canada, the USA, and England agreed with conspiracy beliefs related to the COVID-19 outbreak [4–6]. Conspiracy beliefs about the COVID-19 pandemic were also found to be highly interrelated with a general conspiracy mentality [7–9].

Besides fear and the need for control, anxiety was identified as a psychological aspect contributing to conspiracy mentality. Particularly during the corona crisis, anxiety and also loneliness increased among the general population [5, 10–12] supporting the idea that those variables are important psychological state factors in relation to conspiracy beliefs [5]. Previous studies found anxiety and distress to be related to conspiracy beliefs during the pandemic in the general population, health care workers, and students [5, 13, 14]. Socio-demographic characteristics such as a low social status, low education, and being part of an ethnic minority showed additional associations with conspiracy mentality [3]. Younger individuals and men were more likely to endorse conspiracy beliefs [15]. In addition, subjective feelings of political, economic, and social deprivation were associated with conspiracy mentality [2, 16, 17], hinting at a close interaction of societal and psychological factors for its progression.

However, the influence of specific aspects of personality on conspiracy mentality has remained rather controversial, as previous studies showed mixed findings [16, 18, 19]. For example, associations between openness, (lacking) agreeableness, (lacking) self-esteem, and conspiracist thinking could not be consistently replicated. Still, emotional instability and neuroticism show rather robust associations with conspirational thinking [20], while paranormal beliefs and schizotypy have been found to occur within conspiracist ideation [21–24]. A better understanding of personality functioning might help explain these inconsistencies and better understand its role in conspiracy endorsement.

With the introduction of the DSM-5 Alternative Model for the Assessment of Personality Disorders (AMPD; [25]) it became possible to conduct a dimensional rating of basic psychological capacities. The resulting general indicator of psychological functioning allowed for a severity assessment of personality dysfunction, and hence, can be seen as a requirement for further differentiation of personality disorders where the application of cut-off scores allow for categorical diagnoses [26]. In addition, assessment of (impaired) personality functioning can be used as a stand-alone measure to inform treatment planning and evaluation [27]. This hybrid model of dimensional and categorical measures of personality functioning has also been used in the revision of the related chapter of the ICD-11 [28]. Comparable to the DSM-5 and the ICD-11, the Operationalized Psychodynamic Diagnosis system (OPD; [29]) is another approach consisting of similar constructs of personality functioning. The OPD is a multiaxial system using a semi-structured interview to evaluate a patient’s experience of illness, interpersonal relations, intrapsychic conflicts, and personality structure. In the OPD, the term ‘structure’ is the label for personality functioning which is operationalized by means of Levels of Structural Integration (LSIA; [27, 30]). Several studies have confirmed that assessing LSIA is a valid and reliable measure for personality functioning [30–32]. Personality functioning or ‘structure’ describes a person’s abilities in four domains related to capacities of cognition/perception, regulation, communication, and attachment—each directed toward the self and others—resulting in eight basic structural functions [33].

Although it is a relatively stable construct, recent research has shown that personality functioning is modifiable, e.g., through psychotherapy [34, 35]. To capture changes in personality functioning in a clinical context and make the concept usable for research, the OPD-Structure Questionnaire (OPD-SQ; [36]) and a short form of it (OPD-SQS; [37]) have been developed as self-report instruments [38]. The OPD-SQ is highly correlated with personality functioning according to the DSM-5 [39]. The OPD-SQS assesses three subscales (*self-perception*, *interpersonal contact*, and *relationship model*) derived from the original eight dimensions of the LSIA. The subscale *interpersonal contact* focuses on interpersonal skills associated with aspects of self-uncertainty. It includes self-esteem regulation, making contact, and, communicating affect. *Self-perception* links aspects of the self to emotion regulation skills and includes identity, self-reflection, affect differentiation, and affect tolerance. The subscale *relationship model* includes relationship experiences and corresponding expectations of new relationships. It is conceptually related to the attachment theory, which describes mental representations of relationships that first develop in infancy. Based on these internal representations, expectations about future relationships are built up in the course of life [40]. This subscale includes internalization, self-object differentiation, and realistic object perception [37, 41]. To sum it up, from a psychodynamic perspective, the three subscales describe the level of personality functioning which differs between individuals and is described as more or less well integrated. Individuals with a well-integrated structure and higher abilities on the three subscales have the psychological capacities to develop a coherent self-image, meaning that the cognitions and feelings one has towards oneself are stable and both negative and positive thoughts and feelings are integrated into the self-image. With respect to the three subscales, persons with a well-integrated structure have less difficulty connecting with others and exhibit good levels of self-esteem or self-confidence. They are able to bond with others in stable and healthy relationships and feel integrated into society. They are able to differentiate, tolerate, regulate, and communicate their affects and are capable of self-reflection.. On the other hand, individuals with a less well-integrated structure have difficulty making interpersonal contact, feel excluded and often show self-uncertainty and low self-esteem. They find it difficult to trust others and to establish lasting and stable relationships. Their capacity for self-reflection is low, as is their affect differentiation, affect tolerance, and affect communication [42]. Generally, personality functions also shape psychosocial functioning in daily life and enable a person to adapt to reality in a flexible way. In accordance with Franks and colleagues [43] who defined the view of reality, of oneself, and of in- and out-groups as three of six dimensions involved in conspiracy beliefs, a lower level of personality functioning can be assumed to contribute to the endorsement of conspiracy beliefs. Although, to the authors’ knowledge, there are no previous studies that analyzed personality functioning and conspiracy beliefs, there are findings that various aspects of personality functioning, such as low trust, insecure attachment, emotional instability, and low self-esteem are related to higher conspiracy thinking [16–18, 20].

Understanding the underlying mechanisms of conspiracy mentality regarding socio-demographic, personality and situational psychological aspects could contribute to the development of target group specific information and programs during the pandemic. The aim of this study was to examine the relationship between conspiracy mentality and conspiracy-related beliefs toward the COVID-19 pandemic, accounting for socio-demographic characteristics, personality functioning, and psychological state variables (anxiety and loneliness). We formulated the following research questions:

1. What is the prevalence of conspiracy beliefs and conspiracy-related beliefs toward the COVID-19 pandemic?
2. What are relevant correlates of conspiracy mentality regarding a) socio-demographic characteristics, b) personality functioning, and c) psychological state variables (anxiety and loneliness)?

Further, we hypothesized that:

1. General conspiracy beliefs are positively associated with conspiracy-related beliefs toward the COVID-19 pandemic.
2. A lower level of personality functioning is associated with conspiracy mentality and conspiracy-related beliefs toward the pandemic.
3. Higher levels of anxiety and loneliness are associated with conspiracy-related beliefs toward the pandemic.

## Materials and methods

In cooperation with the independent demography research institute USUMA Berlin, data of a representative sample of the German population (age 14–95) were collected between May, 2^nd^ and June, 29^th^ 2020, employing a random route approach. First, 258 German regional areas were predefined using the reference system for representative studies in Germany provided by the ADM-Sampling-System. Afterwards, the target households within these regional areas were selected following a random route procedure. For multi-person households, one person was randomly selected by means of the Kish selection grid technique. Face to face interviews were carried out following hygiene regulations (wearing facial masks and keeping physical distance). A total of 2,503 participants took part, representing 46.5% of the 5,418 addressed households. By comparisons with the Federal Statistical Office, the sample proved representative for the German general population regarding age, gender, and education.

The study was conducted in accordance with the Declaration of Helsinki and fulfilled the ethical guidelines of the International Code of Marketing and Social Research Practice of the International Chamber of Commerce and the European Society of Opinion and Marketing Research. Prior to being carried out, the surveys were approved by the Ethics Committee of the Medical Faculty of the University of Leipzig. Anonymity in responses was guaranteed and informed consent was obtained from all respondents who indicated willingness to take part in the study. In line with the guidelines of the Working Group German Marketing Institutes and Social Research Practice, it is generally assumed that respondents aged 14 or above are capable of consenting to the use of the information provided in survey research. However, due to the nature of face-to-face studies, at least one parent is usually informed about the content of the study and the selection procedure before the survey begins. To be eligible for survey inclusion, participants had to be at least 14 years of age and have sufficient German language skills.

### Measures

Socio-demographic information such as age, gender, migration background, level of education, and household income were assessed during the interview. Additional information was obtained via a questionnaire which was handed out in a sealable envelope. Subsequently, socio-demographic and questionnaire data were linked. Age was assessed by year and month of birth. To determine gender, participants were asked to choose between male, female, or diverse. If a participant or at least one of her/his parents was born without German citizenship, this individual was defined as someone with a migration background, following the definition of the German Federal Statistical Office [44]. The level of education was assessed according to the German school system. It was recoded as years of schooling for comparability. High education was assumed if a participant attended more than ten years of schooling. Equalized income was calculated according to the OECD guideline by dividing the household income through the square root of people living in the household [45].

Conspiracy mentality was assessed with a short version of the Conspiracy Mentality Scale measuring generic conspiracy beliefs [46] (shortened according to [47]. Participants rated three items (e.g., "Most people do not realize the extent to which our lives are determined by conspiracies hatched in secret.”) on a scale ranging from 1 = “does not apply at all” to 7 = “fully applies”. The sum score thus ranged from 3 to 21. As used and suggested in previous studies, a cut-off score above 12 was used to define a manifest conspiracy mentality (Decker & Brähler, 2020). In our sample, the internal consistency was excellent (ω = .907). Conspiracy-related beliefs toward the COVID-19 pandemic were assessed using two items (e.g., "The true doings behind the COVID-19 pandemic will never come to the light of day.") developed by Schließler and colleagues [17]. The items were rated from 1 = “does not apply at all” to 7 = “fully applies”. The sum score thus ranged from 2 to 14. As used and suggested in previous studies, a cut-off score above 8 was used indicating strong agreement with conspiracy-related beliefs toward the COVID-19 pandemic [3]. In our sample the internal consistency was acceptable (ω = .797).

The short form of the self-report Operationalized Psychodynamic Diagnosis Structure Questionnaire (OPD-SQS; [36]) was used to assess the level of personality functioning. It consists of 12 items with a three-factor structure. The three-factor structure was examined with a good model fit in previous studies [41]. Four items each serve as indicators for this latent dimensions which describe *self-perception (e*.*g*., *“I sometimes experience myself as a stranger”)*, *interpersonal contact (e*.*g*., *“I have been hurt a lot because I was wrong about a person”)*, and *relationship model (e*.*g*., *“I have a hard time connecting with others“)*. Each item is rated from 0 = “does not apply at all” to 4 = “fully applies”. The sum score ranges from 0 = "highest structural level" to 48 = "lowest structural level" [37]. Thus, a higher score indicates impaired personality functions and greater difficulties in *self-perception*, *interpersonal contact*, and *relationship models*. The OPD-SQS showed a good internal consistency with Cronbach’s α = .88 in a clinical and non-clinical sample [37]. In our sample the overall internal consistency was good (ω = .894). With respect to individual subscales, it was acceptable for *interpersonal contact* (ω = .772) and *relationship model* (ω = .788) and good for *self-perception* (ω = .847).

A short version of the Hospital Anxiety and Depression Scale [48], the HADS-6 as a valid and reliable instrument to screen for depressive and anxious symptoms was used [49]. For this study, we only used the three items assessing symptoms of anxiety (e.g., “I am overcome by a fearful premonition that something terrible could happen” [50]. The items were rated from 0 = “most of the time” to 3 = “not at all”. A higher score indicates more anxiety. In our sample the internal consistency was acceptable (ω = .756).

Loneliness was assessed using the German version [51] of the three-item UCLA Loneliness Scale [52]. It consists of three items (e.g., “How often do you feel that you lack companionship?”). Responses are scored from 1 = “never” to 5 = “very often”. The sum score ranges from 3 to 15, with a higher score indicating higher levels of loneliness. It showed good internal consistency in our sample (ω = .823).

### Statistical analysis

Pearson product-moment correlations were conducted to examine the relationship of conspiracy mentality, conspiracy-related beliefs toward the COVID-19 pandemic, personality functioning and psychological state variables. Seven separate multiple linear regression analyses were carried out. Three regression analyses of general conspiracy mentality on socio-demographic variables (model 1), personality functioning (model 2), and psychological state variables (model 3) were conducted. Additionally, four regression analyses of conspiracy-related beliefs toward the COVID-19 pandemic on conspiracy mentality (model control), socio-demographic variables (model 1), personality functioning (model 2), and psychological state variables (model 3) were carried out. Age and income were included as continuous variables, whereas gender (0 = male, 1 = female), migration background (0 = not present, 1 = present), and education (0 = low education, 1 = high education) were used as binary predictors. The sum scores of the OPD-SQS subscales were used as predictors to enable the independent determination of impairment in *self-perception*, *interpersonal contact*, and *relationship model*. Psychological state variables of anxiety and loneliness were used as continuous predictors. Significance for statistical tests was set at *p* < 0.05 (two-sided). Analyses were conducted using SPSS 26. Psychometric analyses were done in JASP version 0.14.1. Effect sizes and correlation coefficients are interpreted following Cohen [53].

## Results

### Sample description

Table 1 gives an overview of the study participants. The mean age of the participants was 46 years and the mean equivalence household income was 2.026 Euro. A migration background was indicated by 15.7% of the participants and 31.5% held at least a German high school diploma, indicating a high level of education in this study. 53.1% of the participants identified as female, 46.9% said they were male, and one person identified as diverse. As only one participant identified as diverse, this person was excluded from further analysis. Using 12 as cut-off score, 38.5% of the participants endorsed a general conspiracy mentality and 41.8% strongly agreed with conspiracy-related beliefs toward the COVID-19 pandemic using 8 as cut-off value.

**Table 1:** Characteristics of the study participants (*N* = 2,503).

	*M*	*SD*	*N*	*%*
Socio-demographic characteristics				
Age in years	45.99	17.77		
Equivalized Income in Euro	2.026,10	1.087,88		
Gender				
Female			1.329	53.1
Male			1.173	46.9
Diverse			1	0.0
High level of education^1^ (yes)			763	31.5
Migration background (yes)			383	15.7
Personality functioning				
OPD-SQS sum score	15.16	8.73		
Self-perception	3.22	3.29		
Contact organization	4.83	3.24		
Relationship model	7.11	3.69		
Psychological state variables				
Anxiety (HADS-6)	2.36	1.98		
Loneliness (UCLA)	6.07	2.54		
Conspiracy				
Conspiracy mentality	10.65	5.42		
Conspiracy mentality (yes)			953	38.5
Conspiracy-related beliefs toward COVID-19	7.62	3.96		
Conspiracy-related beliefs (strong agreement)			1.037	41.8

*Note*.

^1^ At least German high school diploma; socio-demographic characteristics: equivalized income calculated according to the OECD guideline [45], migration background defined following the German Federal Statistical Office [44]. Personality functioning: OPD-SQS = Operationalized Psychodynamic Diagnosis Structure Questionnaire Short, overall score ranges from 0–48, subscale scores range from 0–16 [37]. Psychological state variables: HADS-6 = Hospital Anxiety and Depression Scale, score for anxiety symptoms ranges from 0–9 [49]; UCLA = UCLA Loneliness Scale, score ranges from 3–15 [51]. Conspiracy: conspiracy mentality score ranges from 3–21 with scores above 12 representing a manifest conspiracy mentality, score of conspiracy-related beliefs toward COVID-19 ranges from 2–14 with scores above 8 representing strong agreement [3].

### Correlation analysis

The correlation of conspiracy mentality and conspiracy-related beliefs toward the COVID-19 pandemic was statistically significant and very strong (*r* = .702). Moderate correlations were found for conspiracy mentality and conspiracy-related beliefs toward the COVID-19 pandemic with the OPD-SQS subscale *relationship model*. Weak correlations occurred for the subscales *interpersonal contact* and *self-perception* as well as for anxiety and loneliness. All analyzed correlations were statistically significant (Table 2).

**Table 2:** Correlations of conspiracy mentality and conspiracy-related beliefs toward the COVID-19 pandemic with personality functioning and psychological state variables.

		1.	2.	3.	4.	5.	6.	7.
1.	Conspiracy mentality		.702**	.257**	.183**	.358**	.242**	.186**
2.	Conspiracy-related beliefs COVID-19			.184**	.118**	.308**	.180**	.145**
3	Self-perception (OPD-SQS)				.657**	.567**	.490**	.464**
4	Interpersonal contact (OPD-SQS)					.572**	.419**	.471**
5	Relationship model (OPD-SQS)						.422**	.359**
6	Anxiety (HADS-6)							.522**
7	Loneliness (UCLA)							

***Note***. Significance levels:

*p < .05,

**p < .01,

***p < .001;

OPD-SQS = Operationalized Psychodynamic Diagnosis Structure Questionnaire Short [37]; HADS-6 = Hospital Anxiety and Depression Scale [49]; UCLA = UCLA Loneliness Scale [51].

### Multiple regression analyses

The regression analyses of conspiracy mentality are presented in Table 3. Model 1 including socio-demographic predictors explained 5.1% of the criterion’s variance. Low education, male gender, younger age, and lower income were identified as statistically significant predictors. Migration background showed no significant relationship. Adding the subscale scores of personality functioning in model 2, the explained variance increased to 16.3%. The *relationship model* as subscale of the OPD-SQS showed the strongest association with conspiracy mentality. A higher score and thereby more impairment of personality functions on this scale was statistically predictive for more endorsement of conspiracy beliefs. The OPD-SQS subscale *self-perception* showed a smaller but significant association in the same direction. Lower scores and thereby less impairment of personality functioning on the OPD-SQS subscale *interpersonal contact* were statistically predictive of higher scores of conspiracy mentality. Model 3 including psychological state variables added 1% to the explained variance. Anxiety symptoms as psychological state variable were positively associated with conspiracy mentality, whereas loneliness showed no significant association.

**Table 3:** Linear regression analyses of conspiracy mentality on socio-demographic characteristics, personality functioning, and psychological state variables.

	Model 1		Model 2		Model 3	
	*Beta*	*CI 95%*	*Beta*	*CI 95%*	*Beta*	*CI 95%*
Socio-demographic characteristics						
Gender	-.032	-.79, .09	**-.075** ***	-1.24, -.40	**-.089** ***	-1.39, -.54
Age	**-.113** ***	-.05, -.02	**-.078** **	-.04, -.01	**-.090** **	-.04, -.02
Migration background	.036	-.08, 1.15	.004	-.53, .65	-.003	-.65, .54
High education	**-.215** ***	-2.97, -2.02	**-.171** ***	-2.46, -1.54	**-.174** ***	-2.49, -1.57
Income	**-.098** ***	-.38, -.15	**-.076** **	-.32, -.10	**-.068** **	-.30, -.07
Personality functioning (OPD-SQS)						
elf-perception			**.119** ***	.11, .29	**.079** **	.04, .22
Contact organization			**-.116** ***	-.29, -.11	**-.133** ***	-.31, -.13
Relationship model			**.319** ***	.41, .54	**.296** ***	.36, .51
Psychological state variables						
Anxiety (HADS-6)					**.122** ***	-.20, .47
Loneliness (UCLA)					.014	-.08, .13
Adj. R^2^	.075		.180		.191	

*Note*. Statistically significant predictors are printed in bold. Significance levels:

*p < 0.05,

**p < 0.01,

***p < 0.001;

OPD-SQS = Operationalized Psychodynamic Diagnosis Structure Questionnaire Short [37]; HADS-6 = Hospital Anxiety and Depression Scale [49]; UCLA = UCLA Loneliness Scale [51].

Regression analyses of conspiracy-related beliefs toward the COVID-19 pandemic are shown in Table 4. Controlling for conspiracy mentality, 49.3% of the criterion’s variance was explained. Adding socio-demographic characteristics (model 1), subscale scores of personality functioning (model 2), and psychological state variables (model 3), the explained variance increased around 1% in each case. In model 1, lower education and younger age were statistically significant predictors for conspiracy-related beliefs toward COVID-19. Gender, income, and migration background showed no significant association. In model 2, the subscale *relationship model* showed a positive and significant association with conspiracy-related beliefs toward COVID-19. The OPD-SQS subscale *self-perception* was no longer significantly predictive. The OPD-SQS subscale *interpersonal contact* was negatively and significantly predictive for higher scores of conspiracy-related beliefs toward the pandemic. The psychological state variables (model 3) showed no significant association with conspiracy-related beliefs.

**Table 4:** Linear regression analyses of conspiracy-related beliefs toward COVID-19 on conspiracy mentality, socio-demographic characteristics, personality functioning, and psychological state variables.

	Control		Model 1		Model 2		Model 3	
	Beta	CI 95%	Beta	CI 95%	Beta	CI 95%	Beta	CI 95%
Conspiracy mentality								
	**.702** ***	.49, .54	**.664** ***	.47, .51	**.641** ***	.45, .50	**.645** ***	.45, .50
Socio-demographic characteristics								
Gender			.023	-.05, .42	.014	-.13, .35	.009	-.17, .31
Age			**-.070** ***	-.02, -.01	**-.078** ***	-.03 -.01	**-.073** ***	-.02, -.01
Migration background			**.051** **	.23, .90	**.043** *	.14, .81	.038	.08, .75
High Education			**-.126** ***	-1.3, -.81	**-.126** ***	-1.3, -.81	**-.124** ***	-1.3, -.80
Income			-.004	-.07, .05	-.007	-.08, .05	-.006	-.08, .05
Personality functioning (OPD-SQS)								
Self-perception					-.009	-.06, .04	-.014	-.07, .04
Contact organization					**-.081** ***	-.15, -.05	**-.085** ***	-.16, -.05
Relationship model					**.102** ***	.07, .16	**.104** ***	.07, .16
Psychological state variables								
Anxiety (HADS-6)							-.007	-.09, .06
Loneliness (UCLA)							.026	-.02, .10
Adj. R^2^	.493		.513		.518		.522	

*Note*. Statistically significant predictors are printed in bold. Significance levels:

*p < 0.05,

**p < 0.01,

***p < 0.001;

OPD-SQS = Operationalized Psychodynamic Diagnosis Structure Questionnaire Short [37]; HADS-6 = Hospital Anxiety and Depression Scale [49]; UCLA = UCLA Loneliness Scale [51].

## Discussion

Based on data of a representative survey of the German population, this study examined the associations of socio-demographic characteristics, personality functioning, anxiety, and loneliness with conspiracy mentality and, more particularly, conspiracy-related beliefs toward the COVID-19 pandemic. During the pandemic, the understanding of conspiracy beliefs and their underlying mechanisms are highly relevant because of their relationship with both non-preventive behaviors and refusal of vaccination and also with political attitudes like authoritarianism and other antidemocratic prejudices. As conspiracy mentality is associated with authoritarianism, right-wing populist party preferences and self-localization on the right margin of the political spectrum, it is an important catalyst of radicalization [54]. Thus, even comparatively small numbers of individuals who endorse conspiracy beliefs could undermine health-related efforts and affect the political climate of society.

Using previously determined cut-off values, our results showed that in the general German population, 38.5% reported a general conspiracy mentality and a considerable proportion of 41.5% endorsed the beliefs that the background to the COVID-19 pandemic will never come to the light of day and that the corona crisis has been widely publicized so that only a few people benefit from it, respectively. This prevalence rate is around 10% lower than in online studies conducted during the pandemic in England, North America, and Canada with non-probability samples [4–6]. The prevalence of conspiracy thinking appears to be lower in the general population than in convenience samples, which are likely to be younger and have a higher affinity for the Internet. Comparable studies with representative samples are rare and do not report prevalence rates, such as one study with a representative sample of 11,523 people in nine countries [55]. The findings of Freeman and colleagues [4] had been questioned because of the use of a unipolar scale [56], whereas Leibovitz and colleagues [5] used a bipolar scale. Moreover, we did not assess specific conspiracy beliefs in the context of COVID-19, e.g., about the injection of a microchip with the help of the vaccination or the belief that the coronavirus is a hoax or a bioweapon. Therefore, it seems important to define the items used in the present study not as COVID-19 specific conspiracy beliefs but rather as conspiracy-related beliefs toward the pandemic. Our items were newly developed [17] but closely resembled a validated conspiracy scale [47]. Indeed, our findings show that the newly devised items were strongly correlated with general conspiracy beliefs.

In line with previous research, lower educational attainment, lower income, younger age, and male gender statistically predicted the endorsement of conspiracy beliefs [2, 3, 16, 17]. Lower educational attainment and younger age even stayed predictive for conspiracy-related beliefs toward the pandemic when controlled for general conspiracy mentality. Young individuals seem to be an important target group to be addressed specifically e.g., by using social media channels when planning information or prevention campaigns [57, 58]. As education seems to be a key factor not only in the context of conspiracy mentality in general but also during the current pandemic, more efforts are needed to develop and distribute more easily accessible and understandable information about the pandemic. Participants with a higher level of anxiety were more likely to endorse conspiracy beliefs. In this case it is important to mention that our study does not contribute to an understanding of the causality of this observed effect. A higher level of anxiety might lead to conspiracy beliefs, that serve to reduce anxiety, while conspiracy mentality could also increase anxiety [59]. Contrary to our hypotheses, anxiety showed no statistically significant effect for conspiracy-related beliefs towards the COVID-19 pandemic when controlled for general conspiracy mentality.

Specific subscales of the OPD-SQS were positively, resp. negatively associated with the endorsement of conspiracy beliefs. A higher score on the dimension relationship model was significantly associated with the endorsement of conspiracy mentality and conspiracy-related beliefs toward the COVID-19 pandemic. The relationship model is associated with the ability to bond with others and the expectations of existing and new relationships [37]. Participants with a higher score on this subscale have greater difficulties in their relationships i.e., to trust and feeling being a part of society. The high predictive value of the subscale relationship model seems plausible because the relationship model is conceptually linked to the attachment theory and previous research found an association between an insecure attachment style and conspiracy beliefs [60]. This matches findings about the association between feelings of social exclusion and conspiracy beliefs [61, 62]. The subscale self-perception was positively associated with conspiracy mentality. Thus, low self-reflection, impairment in affect differentiation, and affect tolerance seem to be related to conspiracy mentality. However, self-perception had statistically no predictive value concerning conspiracy-related beliefs toward the COVID-19 pandemic. This might indicate that in times of crises, self-perception becomes less important compared to the relationship to others. The subscale interpersonal contact was negatively associated with conspiracy mentality and conspiracy-related beliefs toward the COVID-19 pandemic. A lower score and thereby less impairment in personality functions concerning interpersonal contact was related to stronger agreement with conspiracy beliefs. This could indicate that it is easier for a person who believes in conspiracies to establish interpersonal contact, get in touch and communicate own affects. On the other hand, the subscale interpersonal contact includes aspects of self-uncertainty and self-esteem regulation. Self-uncertainty and low self-esteem was previously found to be positively associated with conspiracy mentality [15, 18]. Thus, it seems important to further investigate the dimensions of personality functioning and their association with conspiracy beliefs.

### Strengths and limitations

Benefits of the study were the availability of data of a representative face-to-face survey which included information about participants’ personal and socio-demographic characteristics. It also highlighted a construct that has previously not been widely studied in conjunction with conspiracy beliefs and could yield further insights that could inform public health efforts (such as information campaigns and their targeting) in important ways.

However, the results need to be interpreted considering the study’s limitations. For instance, the cross-sectional design does not allow drawing conclusions regarding the direction of associations. The items referring to conspiracy-related beliefs towards the COVID-19 pandemic have not been validated prior to the present investigation. The reported prevalence rates therefore need to be interpreted with caution. However, they offered insights beyond established and more general conspiracy mentality measures and should be validated in further research. The OPD-SQS subscales, which were reported as three factors, were analyzed independently. However, it needs to be mentioned that the correlations of the subscales were high and the authors of the OPD-SQS advise to use the single subscales of the instrument with caution [37]. Further research should use more differentiated questionnaires or structured interviews to assess personality functioning widely.

## Conclusion

The strong associations with personality functioning found in this paper expands empirical research on conspiracy mentality. Beyond socio-demographic vulnerabilities, e.g., lower age and lower education, the dimensions of personality functioning could be considered risk factors for the endorsement of conspiracy mentality. This knowledge might serve to inform prevention programs, interventions, and the communication of public health information. This is especially important during the pandemic because previous studies reported associations between conspiracy endorsement, non-compliance with preventive behaviors, and vaccination refusal [9, 63–65].
